# Identification of Serotype in Culture Negative Pneumococcal Meningitis Using Sequential Multiplex PCR: Implication for Surveillance and Vaccine Design

**DOI:** 10.1371/journal.pone.0003576

**Published:** 2008-10-31

**Authors:** Samir K. Saha, Gary L. Darmstadt, Abdullah H. Baqui, Belal Hossain, Maksuda Islam, Dona Foster, Hassan Al-Emran, Aliya Naheed, Shams El Arifeen, Stephen P. Luby, Mathuram Santosham, Derrick Crook

**Affiliations:** 1 Department of Microbiology, Bangladesh Institute of Child Health, Dhaka Shishu (Children) Hospital, Dhaka, Bangladesh; 2 Department of International Health, Johns Hopkins Bloomberg School of Public Health, Baltimore, Maryland, United States of America; 3 Infectious Diseases and Clinical Microbiology, University of Oxford, Oxford, United Kingdom; 4 ICDDR,B: International Centre for Diarrhoeal Disease Research, Bangladesh, Dhaka, Bangladesh; Columbia University, United States of America

## Abstract

**Background:**

PCR-based serotyping of *Streptococcus pneumoniae* has been proposed as a simpler approach than conventional methods, but has not been applied to strains in Asia where serotypes are diverse and different from other part of the world. Furthermore, PCR has not been used to determine serotype distribution in culture-negative meningitis cases.

**Methodology:**

Thirty six serotype-specific primers, 7 newly designed and 29 previously published, were arranged in 7 multiplex PCR sets, each in new hierarchies designed for overall serotype distribution in Bangladesh, and specifically for meningitis and non-meningitis isolates. Culture-negative CSF specimens were then tested directly for serotype-specific sequences using the meningitis-specific set of primers. PCR-based serotyping of 367 strains of 56 known serotypes showed 100% concordance with quellung reaction test. The first 7 multiplex reactions revealed the serotype of 40% of all, and 31% and 48% non-meningitis and meningitis isolates, respectively. By redesigning the multiplex scheme specifically for non-meningitis or meningitis, the quellung reaction of 43% and 48% of respective isolates could be identified. Direct examination of 127 culture-negative CSF specimens, using the meningitis-specific set of primers, yielded serotype for 51 additional cases.

**Conclusions:**

This PCR approach, could improve ascertainment of pneumococcal serotype distributions, especially for meningitis in settings with high prior use of antibiotics.

## Introduction

*Streptococcus pneumoniae* is the predominant cause of bacterial pneumonia, a leading cause of meningitis, and results in more than 800,000 deaths yearly among children under–5 worldwide [1]. Pneumococcal meningitis in developing countries is often recognized late, resulting in high mortality and a substantial burden of long-term disability among survivors. The prospects for prevention of pneumococcal disease and averting deaths are now substantial through mass vaccination using conjugate pneumococcal vaccines [2]–[6].

In many countries, particularly resource poor countries, however, implementing an effective vaccine programme is challenging both in terms of cost and for determining the true burden of diseases, because many pneumococcal meningitis cases are culture negative [7], [8]. Thus, it is not possible to know which serotype caused the disease in most cases, and in turn which capsular antigens should be incorporated into the vaccine. Of the 90 different possible serotypes, disease is caused by a restricted set that can differ markedly between countries. The currently licensed vaccine, a 7-valent pneumococcal conjugate vaccine (PCV7), is based on the predominant serotypes causing invasive disease in North America, and, while it affords protection to >80% of invasive pneumococcal cases in these settings, it is predicted to protect against only 60% and 40% of cases in Africa and Asia, respectively [9]. Moreover, recent observations in Bangladesh [10]–[12] and surrounding countries [13], [14] suggest that this 7-valent formulation may only protect against 25% to 40% of invasive pneumococcal disease among under 5 year-old children.

Although coverage of pneumococcal serotypes with the PCV7 and other upcoming vaccines is estimated to be lowest in Asia, the calculations are based on a small number of isolates. This paucity of data arises from the following factors: i) CSF samples are not routinely collected in Asia, even if the clinical suspicion is very high, ii) few laboratories successfully culture pneumococcus in cases of pneumococcal disease and iii) even fewer laboratories have the resources to serotype the isolates.

A major limitation of our understanding of pneumococcal serotype distribution is that most cases, including meningitis, remain culture negative due to widespread use of antibiotics prior to presenting to the hospital. This limits the survey of capsular serotype distribution of pneumococcal invasive disease and also potentially biases towards antibiotic resistant strains/serotypes. A molecular approach that identifies a pneumococcal etiology and its serotype among both culture positive and culture negative cases would offer greater precision in pneumococcal serotype surveillance and in vaccine design.

Presently, serotyping of pneumococci is dependent on isolation of the organism followed by serological determination by quellung reaction (capsular swelling) [15]. The high cost of antisera, the requirements for technical skills and the complexity in interpretation of results are major hindrances of this procedure, thus further limiting its use in resource poor environments. To overcome these barriers and simplify capsular typing of pneumococci, different initial molecular approaches were attempted; however, they were of limited value and could not be easily used for comprehensive surveillance [16]–[21].

The availability of sequences of the *cps* loci for all 90 known pneumococcal serotypes (http://www.sanger.ac.uk/Projects/S_pneumoniae/CPS/) has facilitated the design of primers for a sequential multiplex polymerase chain reaction (PCR) for capsular serotyping of pneumococci [22], [23], though the primer selection and their arrangement for multiplexing was optimized based on the capsular serotype distribution found in the USA. Despite this geographic limitation in its design, this scheme has been used successfully in field studies on isolates from Brazil and South Africa [24], [25]. There is an urgent need to redesign and optimize the test for the divergent serotypes encountered in Bangladesh and neighboring countries [10]–[14].

In this study we aimed to a) modify the design of the sequential multiplex PCR method of Pai *et al*. [23] to optimize the scheme for the capsular serotype distribution likely to occur in Bangladesh, b) validate the scheme in Bangladeshi strains isolated from meningitis and non-meningitis cases, c) apply the scheme to detect capsular serotype directly from cerebrospinal fluid (CSF) specimens of culture positive and culture negative cases of pneumococcal meningitis and d) to compare the cost and laboriousness of two serotyping methods.

## Methods

### Setting

The study was carried out at Department of Microbiology, Dhaka Shishu Hospital (DSH), the largest pediatric hospital in Bangladesh that provides both primary and tertiary care. DSH has 450 beds of which 50% provide free care, including investigations, food and essential first-line medicines/antibiotics. Nonpaying beds are reserved for those who cannot afford to pay for their care. Lumbar puncture is routinely done for all suspected cases of meningitis, and CSF specimens are analyzed in the Department of Microbiology.

The department is conducting laboratory surveillance for bacterial etiology of meningitis since 1993 [10], [11], [26]–[28]. In addition, since 2004, the surveillance for invasive pneumococcal diseases is extended to a network of 7 hospitals through a PneumoADIP sponsored study [29] (Figure S1).

### Serotyping of strains by Quellung reaction

Pneumococcal strains were serotyped by the capsular swelling procedure (quellung reaction) with anti-pneumococcal omni, pool, type or group, and factor sera (Statens Serum Institut, Copenhagen, Denmark) as described previously [26], [30].

### Primer design and validation

Five new primers not included in the study of Pai *et al.* [23] were designed based on serotype distribution of pneumococcus isolates from pneumonia and meningitis cases enrolled at 7 hospitals of Bangladesh during January 04 to December 06 [29], and 2 other primers (serogroup 12 and 15) were redesigned to yield product sizes appropriate for the reformulated multiplex PCR scheme. All the primers targeted the *wzy/wzx* region of the capsulation locus [22], [23], using sequences downloaded from http://www.ncbi.nlm.nih.gov/Genbank for each of the capsular serotypes/groups; for accession numbers, see Table 1.

**Table 1:** New oligo-nucleotide primers designed in this study.

Serotype	Accession no.		Sequence	Gene location at *cps* loci	Primer position	Product size
2	CR931633.	F	GTCATTGTTACGATTAGTTTCGATAGTTGAGG	*wchI* 11162–12319	11221–11601	381
		R	AATTCAATTCCTAAGTCCTCTTCCATAAACTC			
45	CR931718	F	GTTTAATGGCTGATGAAGTTATTATTGTTG	*wcxS* 10753–11673	10832–11069	238
		R	TTTACCATCAGTGAAATTTTATCTTTGTTC			
24 A/F	CR931686.	F	TCTCAACCAAGATACAGATTTTGATTTTACTC	*wcxI* 12794–13717	12868–13553	686
		R	TATAAACCTTTAGTAAACACTCTGCTTGATCG			
23B	CR931684	F	TTGTTAGTGGTATTAAATTGGGGACTACTAGG	*wzx* 12455–13843	12938–13145	216
		R	ATACCTATCTGAAGTGTTATTAACCCACCAAC			
29	CR931694	F	ATTATCTCGGATCAAACAATTCTTTTGTAAAC	*wzy* 6231–7412	6970–7228	259
		R	AACGCTAACATTAAAACTAGAACGAGTAAACC			
15B/C	CR931664.	F	AGGAATCAGATATTATCATTACTCATGGTG	*wchK-wzy* 6612–8277	6799–7294	496
		R	TCATGACCCATAGAACTATATAAAAAGACG			
12 A/F	CR931658	F	ACTCTTCCAAATTCTTATGCTTTTATTGATTC	*wzy* 11091–12302	11136–11778	656
		R	ATGAATGAGAAAAGGAACTTAAAATTCATAGC			

Newly designed primers were tested for their specificity and cross reactivity against the quellung reaction as gold standard. A full panel of available 56 different serotypes was subjected to amplification. For any discrepant PCR results, we re-confirmed serotype by quellung reaction and re-adjusted the PCR scheme to achieve optimal conditions to resolve these discrepancies.

### Isolates for primer validation

Three hundred and seventy six invasive pneumococcal isolates of 56 different serotypes (1, 2, 3, 4, 5, 6A, 6B, 7A, 7C, 7F, 8, 9N, 9V, 10A, 10F, 11A, 11B, 12A, 12B, 12F, 13, 14, 15A, 15B, 15C, 15F, 16F, 17F, 18A, 18B, 18C, 18F, 19A, 19F, 20, 21, 22A, 22F, 23A, 23B, 23F, 24A, 24B, 24F, 25F, 29, 31, 33B, 33F, 34, 35A, 35B, 35F, 38, 45, 48) were used to validate the new primers. Of these, 257 strains were collected from Bangladesh; 141 were isolated at DSH and 116 were obtained from the 6 hospitals participating in the extended surveillance. Others were received from Oxford University, UK (N =  92), Centers for Disease Control and Prevention, USA (N = 15) and Novartis laboratory, Italy (N = 12), kindly provided by Drs. Derrick Crook, Bernard Beall and Michele Barocchi respectively.

### Primer sets for sequential multiplex testing

The primers were incorporated into 7 sets for sequential testing based on the descending prevalence of pneumococcal isolates occurring from January '04 to December '07. Depending on the ranking of capsular types among pneumonia/sepsis and meningitis cases, the panel of 7 sets of primers was further modified to specifically accommodate the different serotype distributions in these two different disease syndromes [11], [26], [29].

### Cases for CSF specimens

**Figure 1:**
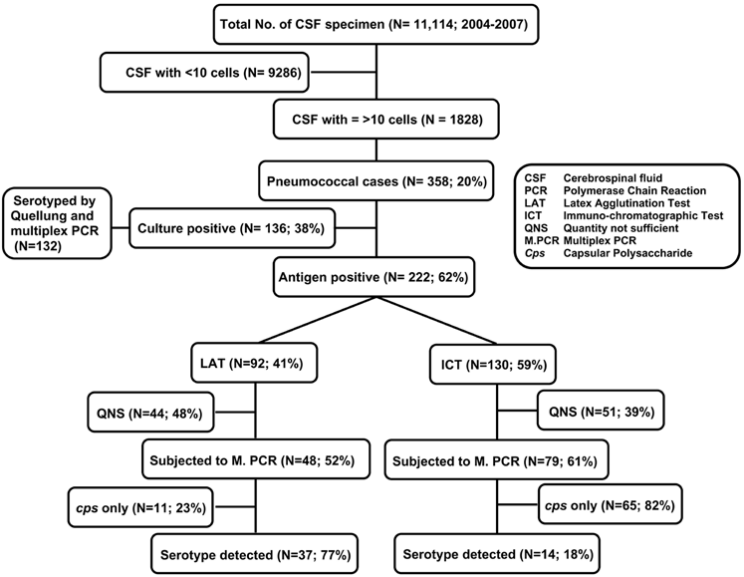
Study profile of CSF specimens with results.

During 2004–2007, 11,114 CSF specimens were received at the Department of Microbiology, DSH, for analysis. Of these, 1,828 had ≥10 cell count/mm^3^, and considered as the cases of meningitis. Pneumococcal meningitis cases (N = 358) were defined based on isolation of the organism or detection of pneumococcal antigen, either by latex agglutination test (LAT; Wellcogen Bacterial Antigen Kit; Remel Europe Ltd, Kent, UK) or immunochromatographic test (ICT) (Binax NOW *Streptococcus pneumoniae* test, Binax Inc., Portland, ME, USA) from CSF (Figure 1).

### PCR method

#### Isolates

PCR reactions for pneumococcus were performed as described by Pai *et al.* [23] with minor modifications. In brief, multiplex PCR was done in 25 µl reaction volume containing 12.5 µl Qiagene Hot Start multiplex PCR master mix with 2.5 µl of equimolar (0.2 µM) primer mix, 2 µl of boiled bacterial suspension and 8 µl of distilled water. PCR tubes were placed in a thermalcycler (DNA Engine, BioRad, Hercules, California, USA) programmed for initial denaturation at 95°C for 15 min followed by 30 cycles of amplification at 94°C for 45 seconds, annealing at 56°C for 1 min 30 seconds, and extension at 72°C for 1min 40 seconds with final extension at 72°C for 10 min. PCR products were electrophoresed in a 2% agarose gel at 90 volts for 150 min, stained with ethidium bromide and visualized by transillumination (Ultra-Violet Products ltd, Upland, California, USA).

#### CSF specimens

Detection of serotype-specific sequences of pneumococcus in CSF specimens was initially standardized and validated by using the culture positive specimens, keeping the respective isolate as control. Based on initial difficulties in amplifying the serotype sequences in culture positive CSF specimens, possibly due to inhibitory substances [31], the specimens were treated with Proteinase K (Sigma-Eldrich, Steinheim, Germany) and precipitated by ethanol to remove inhibitors [32]. The PCR reaction mixture for CSF was equivalent to the mixture for strains, except that 10 µl of CSF was added instead of bacterial lysate and H_2_O. For all PCR reactions, primer for universal pneumococcal genome (*cps*) was incorporated as the control. While running the culture-negative CSF specimens, isolates of known serotypes were included as a built-in positive control for respective multiplex primer sets.

#### Quality assurance

In brief, for quellung reaction, every 10^th^ pneumococcal isolate was cross checked by an independent and blinded person. In case of PCR, a set of 5 isolates of known serotype, were tested by PCR in a blinded way. This was done twice a year by using two different set of randomly selected isolates.

#### Reagent costs and work load

Cost of typing was calculated based on the number of isolates (n = 219) typed in the last 3 years as the antisera generally expires after 3 years from the time of arrival at the laboratory. For quellung reaction, the cost was calculated for one vial of each antisera specifically needed for typing of our isolates. The cost of PCR-based typing included the price of consumables, disposables and quality assurance required for typing the same number of strains. Personnel and capital equipment costs were not considered in the calculation for either of the methods.

Work load was determined on the basis of reactions needed specifically for serotyping of pneumococcal isolates of this study. For capsular swelling method, cumulative reactions needed for pools, groups and types were considered. On the other hand, for PCR typing, multiplex reactions required to identify all the serotypes were added.

### Statistical methods

All the data were entered in Epi-data and analyzed by Epi Info 6.02. P-values were calculated using Statcalc calculator of Epi Info 6.02.

### Ethics

Strains and specimens used in this study were obtained from patients enrolled in different studies. All of them were approved by the Ethical Review Committee (ERC) of Bangladesh Institute of Child Health, DSH. The studies were also approved by the ERC of the collaborative organizations; Johns Hopkins University, USA and International Centre for Diarrhoeal Disease Research, Bangladesh (ICDDR,B).

## Results

**Figure 2:**
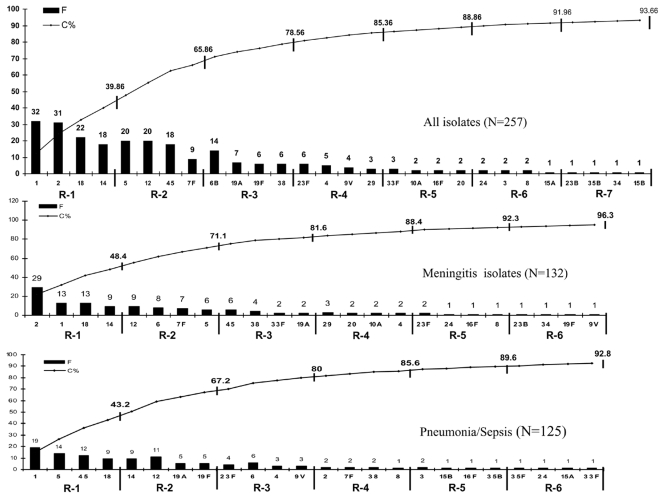
Multiplex PCR schemes and their coverage for Pneumococcal serotypes of Bangladesh

**Figure 3:**
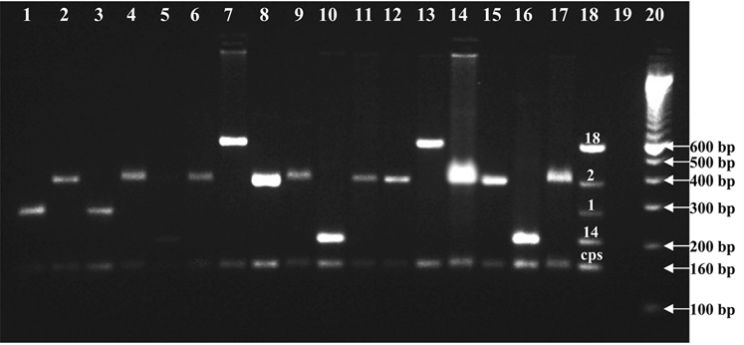
Amplified serotype (1, 2, 14 & 18) genome from culture negative CSF specimens by 1st reaction of multiplex PCR scheme. Lane 1–10 & 14–17: Culture negative latex Positive CSF, Lane 11–13: Culture negative ICT Positive CSF, Lane 18: Positive Control Containing amplified cps gene and genome of 4 known isolates of reaction 1, L 19: Reagent control–reagents devoid template, L 20: 100bp ladder.

The 5 newly designed primers showed 100% specificity when tested using individual isolates of each of 56 different serotypes; there were no discrepant results. The sequential multiplex PCR scheme (Figures 2 and 3), as expected, identified the capsular serotype of isolates in decreasing order of frequency. The first of 7 multiplex reactions yielded the capsular serotype of 40% of strains and, by the end of reaction 4, it reached to 85%. The capsular serotype of 94% of isolates could be determined when using all 7 multiplex reactions (Figure 2A). The initial multiplex design identified the serotype of 31% (N = 39/125) and 48% (N = 64/132) of pneumonia/sepsis and meningitis isolates, respectively (p = 0.007). By redesigning the multiplex scheme specifically for pneumonia/sepsis or meningitis, the capsular serotype of 43% and 48% of isolates could be identified with the 1^st^ reaction, and 86% and 88% of isolates by the 4^th^ reaction, respectively (Figure 2B, 2C).

**Figure 4:**
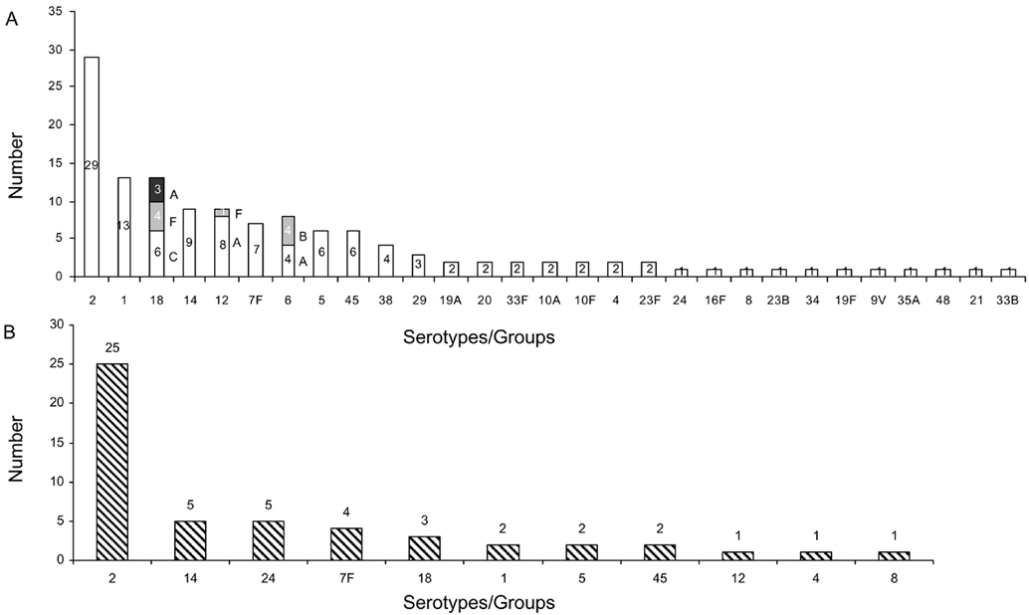
Distribution of capsular types among A) Isolates and B) Culture negative meningitis cases.

During January 2004 to December 2007, there were 358 (20%; 358/1,828) cases of pneumococcal meningitis enrolled at DSH; however, an isolate was grown from only 136 (38%; 136/358) cases. For the other 222 (62%; 222/358) culture-negative cases, the etiology was determined either by LAT (41%; 92/222) or Binax (59%; 130/222). Of these 222 cases, 127 (48 and 79 positives based on LAT and ICT testing, respectively) had sufficient remaining CSF for performing sequential multiplex PCR. PCR yielded the capsular serotype for an additional 51 (40%; 51/127) cases, although the *cps* was positive for all 127 cases. The yield of positive capsular serotype identity by PCR was higher (p<0.001) from the LAT positive cases (77%, 37/48) than from LAT-negative ICT-positive cases (18%, 14/79) (Figure 1). The proportional distribution of PCR-detected serotypes from culture negative cases was similar to the serotype distribution of isolates identified from CSF cultures, except for serotypes 1 and 24, which ranked 2nd and 23rd, respectively, among the cultured isolates, compared to 6th and 3rd, respectively, among the serotypes detected from culture negative cases by PCR (Figure 4A, 4B). Vaccine coverage for serotypes of culture positive meningitis cases by the existing PCV-7 and the upcoming 10 and 13-valent vaccines were 18%, 33% and 42%, respectively. On the other hand, coverage for PCR detected serotypes from culture negative cases was 18%, 33% and 33%, in that order. Coverage of the 3 vaccine formulations for all meningitis-causing serotypes (detected by any method) was 18%, 37% and 40%, respectively.

The calculated cost for serotype determination of 219 strains, collected in 3 years time were US$22,640 for quellung reaction and US$9,688 for PCR method.

Conventional serotyping by quellung reactions required 2,466 reactions (1,186 for pools, 880 for groups and 400 for types). In contrast, PCR based typing needed 688 PCR reactions.

## Discussion

New formulations of pneumococcal conjugate vaccines with higher valency (i.e. 10 and 13 serotypes), aiming for higher coverage and wider use, will soon become available. Recently, Advance Market Commitment (AMC) (http://www.vaccineamc.org/) announced the hope of customizing a pneumococcal vaccine with optimal coverage for all regions, based on region and/or country-specific serotype data, and introducing it promptly in developing countries, including South Asia. To achieve this, the World Health Organization and GAVI's PneumoADIP are working through a Global Serotype Project (GSP) to estimate the burden of serotype-specific pneumococcal disease, by region, sub-region or country, and finally to devise an appropriate target product profile.

Recent use of PCR-based typing has expanded our knowledge of serotype distributions; however, the prevalence of serotypes by disease syndrome and locale is still lacking from many regions. To address that, we designed new primers and rearranged the sequential multiplex PCR reaction scheme according to the predicted distribution of pneumococcal serotypes in Bangladesh.

The 1^st^ reaction of the multiplex PCR scheme (Figure 2) reported here could detect the serotype of up to 40% of invasive pneumococcal cases. This contrasts to the 1^st^ reaction of the schemes designed for use to identify serotypes of major importance in the USA, Africa and Latin America [23]–[25], which would detect only 9%, 18% and 19% of the same Bangladeshi isolates, respectively, indicating the wide differences in distribution of serotypes by geography. Further, Bangladeshi serotypes are also divergent as indicated by lower (66%) cumulative yield from the 1^st^ two multiplex reactions specifically designed for the respective countries, compared to the serotypes of Africa (82%) and Latin America (80%) [24], [25]. The serotype coverage of US strains by multiplex PCR reactions are not shown as the distribution in the USA has been dramatically changed following introduction of the 7-valent conjugate vaccine.

The 1^st^ multiplex reaction following initial customization of the Bangladesh-specific battery of multiplex PCR reactions detected a lower proportion (31%) of pneumonia/sepsis isolates compared to meningitis cases (48%; p = 0.007), mainly due to the excess of serotype 2 among the meningitis cases. Rearrangement of the multiplex reaction schemes accordingly improved the detection of pneumonia/sepsis isolates from 31% to 43% in first reaction. This shows the relative simplicity of this approach to optimizing the multiplex reaction for specific needs, whether geographical or for specific disease syndromes.

In recent years, sensitive diagnostic tests like LAT and Binax have substantially improved measurement of the burden of pneumococcal meningitis [7], specifically in areas where pre-admission use of antibiotics is common such as Asia [8]. However, these tests do not provide capsular serotype information, specifically for culture negative cases. In this report, we identified 358 cases of pneumococcal meningitis among which only 38% (136/358) yielded an isolate; the rest (62%) were identified either by LAT (41%; 92/222) or ICT (59%; 130/222). The sequential multiplex PCR scheme was shown to readily identify the capsular serotype not only of isolates but also directly from CSF samples of culture-negative cases, which was not possible with the Quellung method. The PCR scheme revealed the capsular serotype of an additional 51 cases among 127 available culture-negative specimens, which is equivalent to 38% (51/136) of the culture-positive cases accumulated in the past 48 months. Isolation of this number of strains and identification of their serotype distribution by conventional methods would take about 18 additional months of surveillance and processing of 4,168 specimens, representing a substantial savings of time and money.

Cost of conventional Quellung-based capsular serotyping is 2.4 times more than PCR-based typing, considering the consumables only. In respect of work load, quellung needs about 4 times more number of reactions than PCR. It needs to be mentioned that typing by quellung reaction is more laborious and requires more person time, in comparison to PCR-based typing, even for the same number of reactions [24], [25]. Further, PCR-based serotyping can incorporate built-in controls for quality assurance (QA) of the whole procedure (Figure 3). Nonetheless, capsular serotype data on 171 pneumococcal meningitis cases could not be revealed. Among them, 95 had insufficient specimen to test by PCR, and in another 76 cases sequential multiplex PCR failed to identify the capsular serotype despite samples being positive for capsular elements (i.e. *cps*). Failure of the reaction in these cases (N = 76) could be due to one or more of the following factors: i) insufficient PCR template concentration, ii) presence of residual inhibitory factors, and/or iii) disease caused by uncommon serotypes for which primers are not yet designed and thus omitted from the sequential multiplex PCR (e.g. 10F, 19B, 23A, etc.). It is important to address all these issues to improve the performance and yield of PCR-based capsular serotyping of culture-negative specimens. Lack of sufficient CSF is a common problem, particularly when derived from clinical services; this could be addressed, however, by development of an algorithm of tests relevant to routine patient care. For example, the specimen volume required could be minimized by centralizing all biochemical and microbiological testing in one place, for example the microbiology laboratory, and by testing specimens first with ICT rather than immediately applying both LAT and ICT tests.

In addition to increased cost effectiveness, simplicity and built-in QA of the PCR-based method, multiplex PCR also has the potential to reveal a different distribution of serotypes circulating in the population compared to culture-positive cases. In this study, serotype 24 ranked 23^rd^ in culture-positive cases but ranked 2^nd^ among the culture-negative cases. On the other hand, serotype 1 ranked 2^nd^ in culture-positive cases in contrast to its 6^th^ position among culture-negative cases (Figure 4A, 4B). The difference in serotype distribution of culture-negative cases marginally impacted the overall serotype distribution, possibly because the number of cases was small in comparison to the total number of isolates (51 Vs 136). However, the effect may have been conspicuous if serotype information was revealed from all the culture-negative cases. This could have major impact on formulating the valency of region-specific conjugate vaccines.

The findings reported here are proof-of-principal for value of sequential multiplex PCR capsular serotyping for determination of regional serotype distribution and specifically among cases of pneumococcal meningitis. There is ample scope for improving the yield of the method, by making it more sensitive and adding more primers to allow further differentiation of serogroups, specifically 6A/6B, 12A/12B and 18A/18B/18C. The approach has an important limitation as it will solely increase serotype information from meningitis cases. This shortcoming is critical to keep in mind, as pneumonia serotypes are somewhat different and it causes more deaths than meningitis. Nonetheless, to minimize this, we are extending our work to serotyping of cases with culture-negative and antigen-positive blood culture bottles [33], [34] and pleural fluids. Despite the limitations, the application of the approach described here will offer precise surveillance data on capsular serotype in many more settings by eliminating the complexity of pneumococcal serotyping and the impact of prior antibiotic.

## Supporting Information

Figure S1 Location of 7 Hospitals participating in the invasive pneumococcal disease surveillance network of Bangladesh. 1. Dhaka Medical College Hospital (N = 11); 2. Dhaka Shishu Hospital (N = 141); 3. Shishu Shasthya Foundation (N = 27); 4. Shalimullah Medical College Hospital (N = 10); 5. Kumudini Medical College Hospital (N = 50); 6. Chittagong Ma O Shishu Hospital (N = 10); 7.Chittagong Medical College Hospital (N = 8). (N = Number of strains obtained from network hospitals and used for the primer validation.)
(0.79 MB TIF)
