# Supplementary figures and images for: Identification of Serotype in Culture Negative Pneumococcal Meningitis Using Sequential Multiplex PCR: Implication for Surveillance and Vaccine Design
Source: PLoS One. 2008 Oct 31;3(10):e3576. doi: 10.1371/journal.pone.0003576 (PMC2571985; doi:10.1371/journal.pone.0003576)

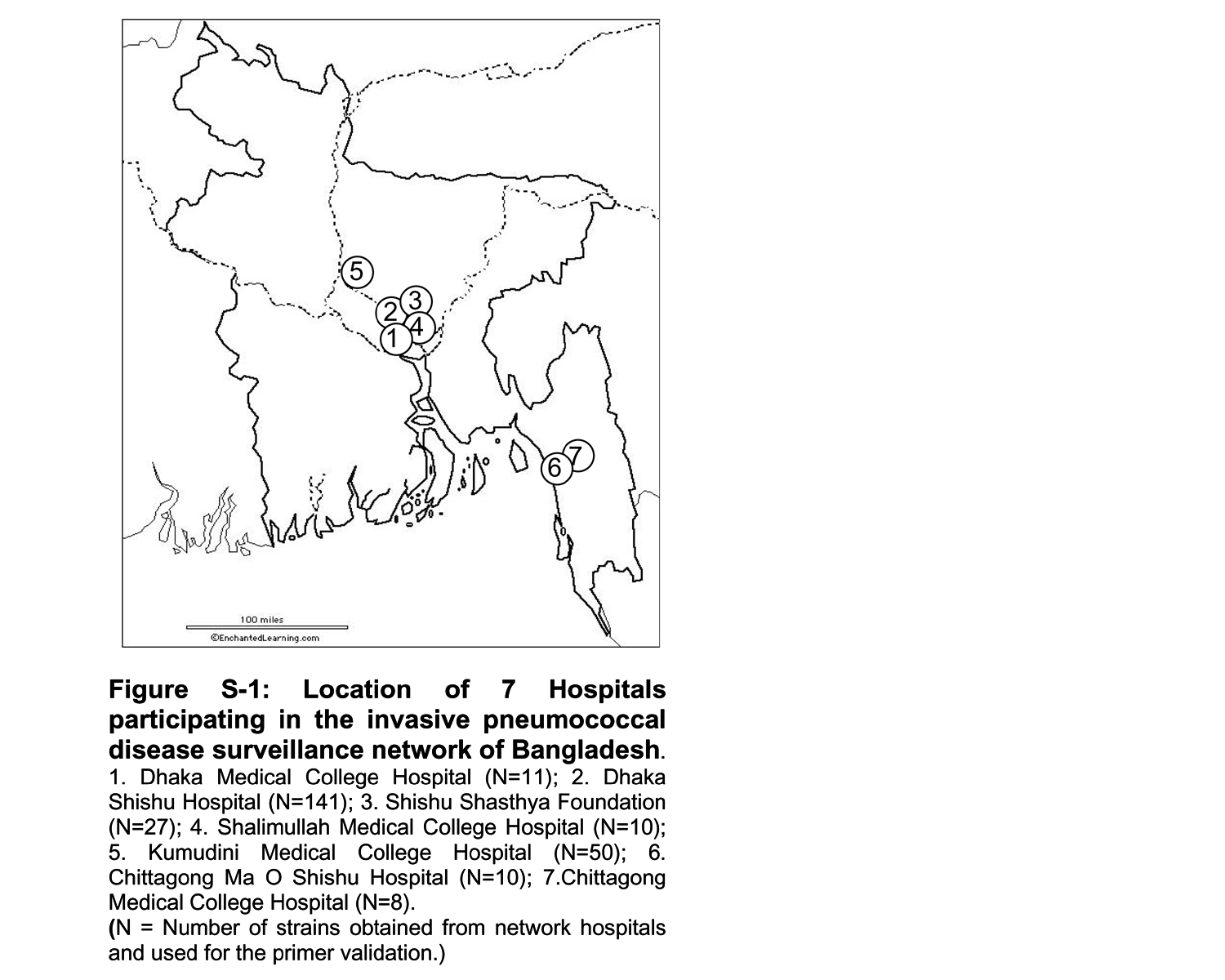

Supplement: Figure S1 — Location of 7 Hospitals participating in the invasive pneumococcal disease surveillance network of Bangladesh. 1. Dhaka Medical College Hospital (N = 11); 2. Dhaka Shishu Hospital (N = 141); 3. Shishu Shasthya Foundation (N = 27); 4. Shalimullah Medical College Hospital (N = 10); 5. Kumudini Medical College Hospital (N = 50); 6. Chittagong Ma O Shishu Hospital (N = 10); 7.Chittagong Medical College Hospital (N = 8). (N = Number of strains obtained from network hospitals and used for the primer validation.) (0.79 MB TIF) [file pone.0003576.s001.tif]
